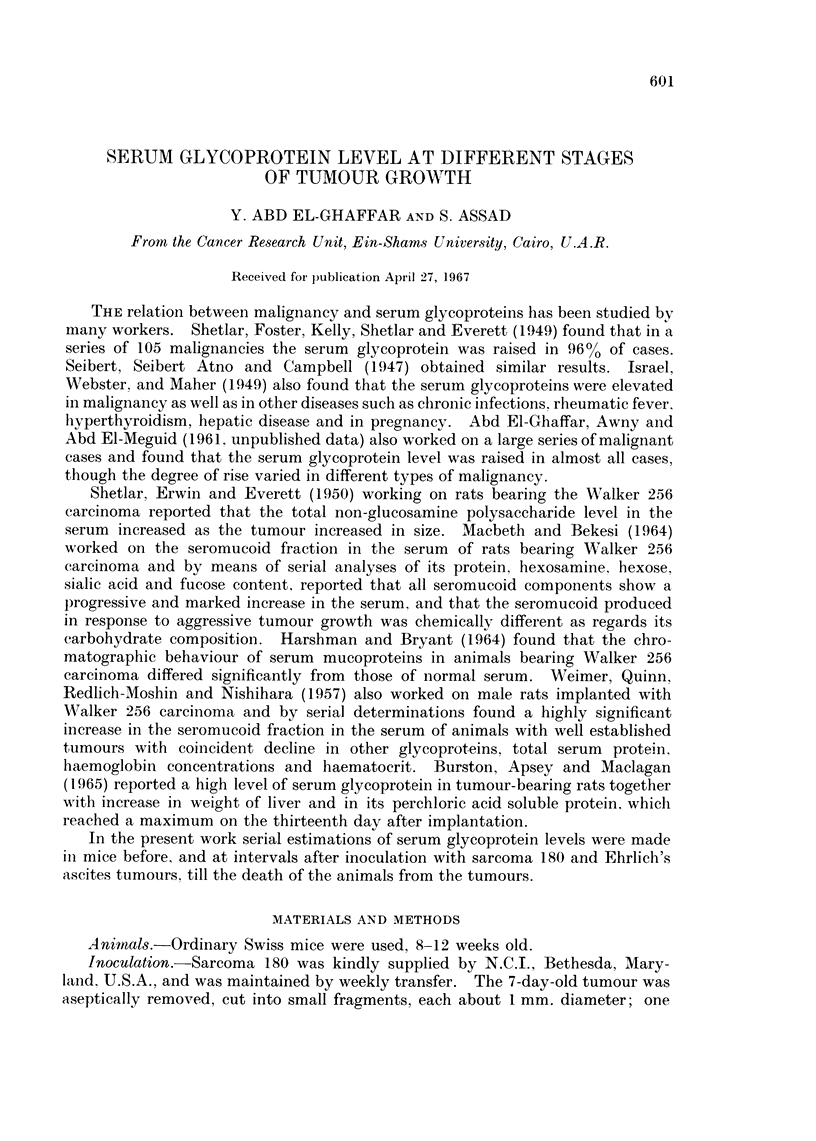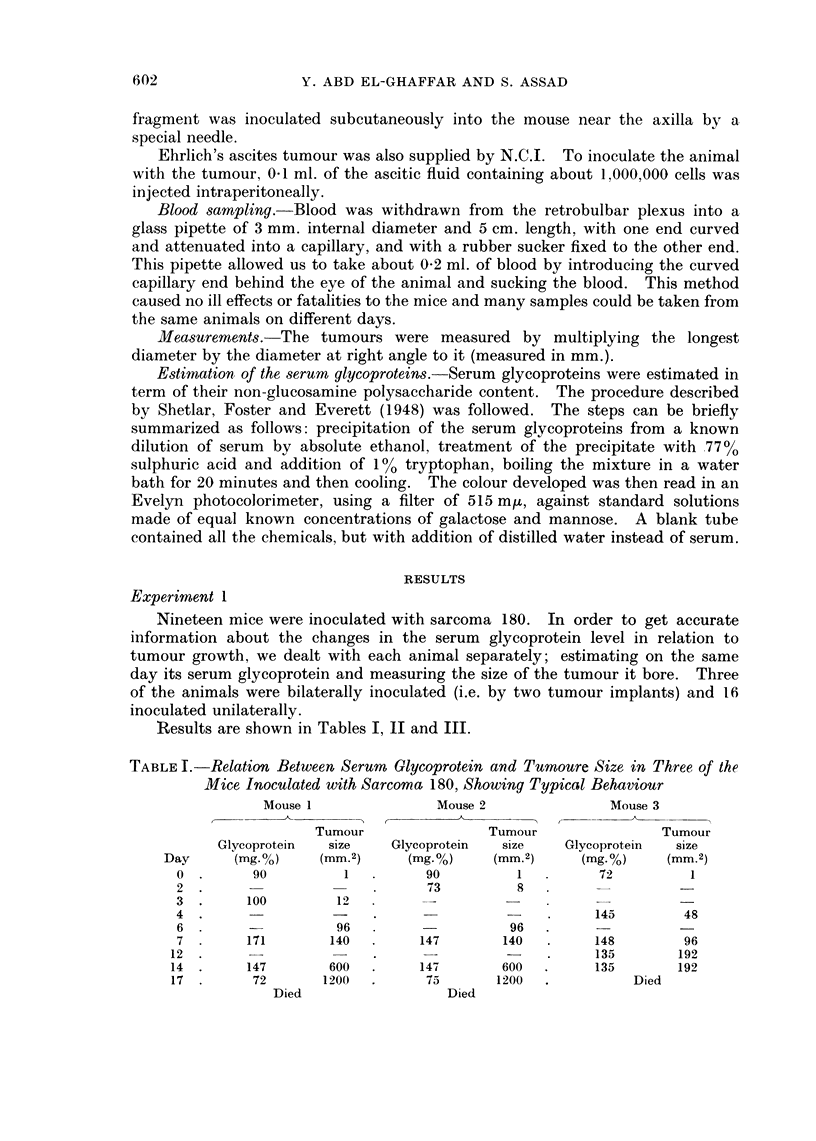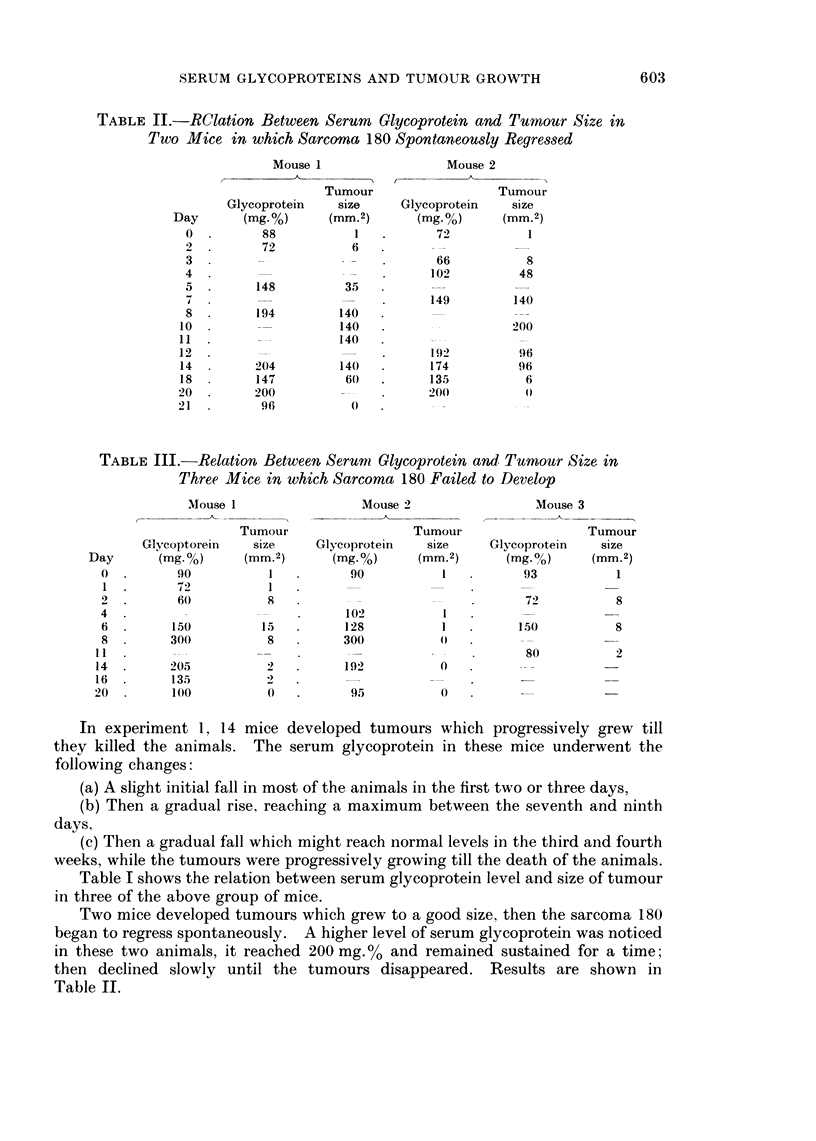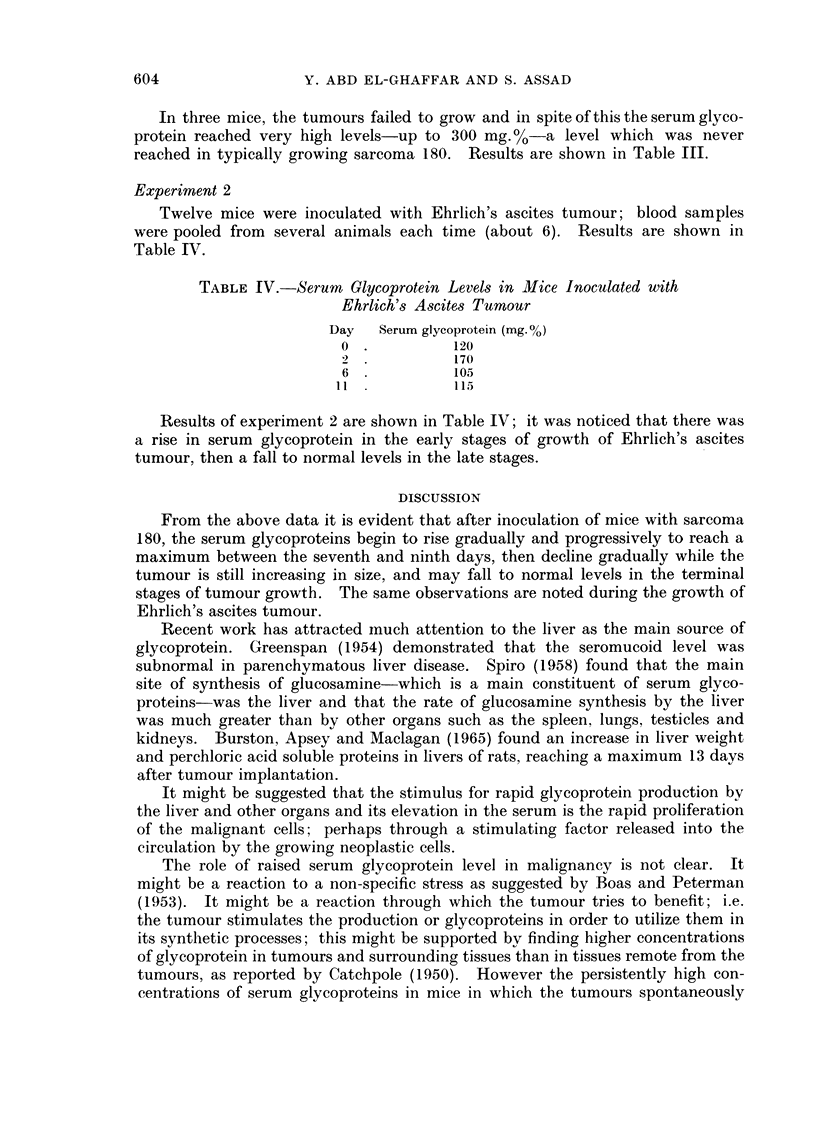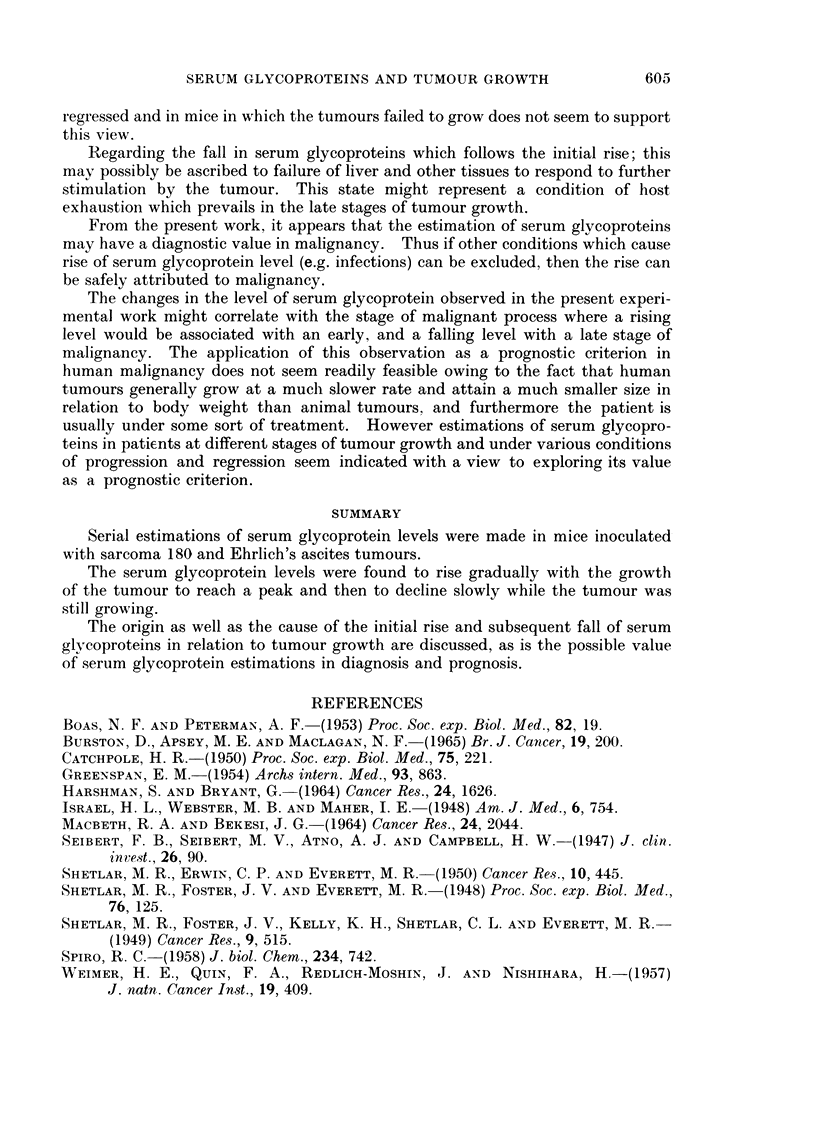# Serum glycoprotein level at different stages of tumour growth.

**DOI:** 10.1038/bjc.1967.70

**Published:** 1967-09

**Authors:** Y. A. El-Ghaffar, S. Assad


### SERUM GLYCOPROTEIN LEVEL AT DIFFERENT STAGES OF TUMOUR GROWTH

Y. ABD EL-GHAFFAR AND S. ASSAD

*From the Cancer Research Unit, Ein-Shams University, Cairo, U.A.R.*

Received for publication April 27, 1967

THE relation between malignancy and serum glycoproteins has been studied by many workers. Shetlar, Foster, Kelly, Shetlar and Everett (1949) found that in a series of 105 malignancies the serum glycoprotein was raised in 96% of cases. Seibert, Seibert Atno and Campbell (1947) obtained similar results. Israel, Webster, and Maher (1949) also found that the serum glycoproteins were elevated in malignancy as well as in other diseases such as chronic infections, rheumatic fever, hyperthyroidism, hepatic disease and in pregnancy. Abd El-Ghaffar, Awny and Abd El-Meguid (1961, unpublished data) also worked on a large series of malignant cases and found that the serum glycoprotein level was raised in almost all cases, though the degree of rise varied in different types of malignancy.

Shetlar, Erwin and Everett (1950) working on rats bearing the Walker 256 carcinoma reported that the total non-glucosamine polysaccharide level in the serum increased as the tumour increased in size. Macbeth and Bekesi (1964) worked on the seromucoid fraction in the serum of rats bearing Walker 256 carcinoma and by means of serial analyses of its protein, hexosamine, hexose, sialic acid and fucose content, reported that all seromucoid components show a progressive and marked increase in the serum, and that the seromucoid produced in response to aggressive tumour growth was chemically different as regards its carbohydrate composition. Harshman and Bryant (1964) found that the chromatographic behaviour of serum mucoproteins in animals bearing Walker 256 carcinoma differed significantly from those of normal serum. Weimer, Quinn, Redlich-Moshin and Nishihara (1957) also worked on male rats implanted with Walker 256 carcinoma and by serial determinations found a highly significant increase in the seromucoid fraction in the serum of animals with well established tumours with coincident decline in other glycoproteins, total serum protein, haemoglobin concentrations and haematocrit. Burston, Apsey and Maclagan (1965) reported a high level of serum glycoprotein in tumour-bearing rats together with increase in weight of liver and in its perchloric acid soluble protein, which reached a maximum on the thirteenth day after implantation.

In the present work serial estimations of serum glycoprotein levels were made in mice before, and at intervals after inoculation with sarcoma 180 and Ehrlich's ascites tumours, till the death of the animals from the tumours.

### MATERIALS AND METHODS

*Animals.*—Ordinary Swiss mice were used, 8–12 weeks old.

*Inoculation.*—Sarcoma 180 was kindly supplied by N.C.I., Bethesda, Maryland, U.S.A., and was maintained by weekly transfer. The 7-day-old tumour was aseptically removed, cut into small fragments, each about 1 mm. diameter; one fragment was inoculated subcutaneously into the mouse near the axilla by a special needle.

Ehrlich's ascites tumour was also supplied by N.C.I. To inoculate the animal with the tumour, 0·1 ml. of the ascitic fluid containing about 1,000,000 cells was injected intraperitoneally.

*Blood sampling.*—Blood was withdrawn from the retrobulbar plexus into a glass pipette of 3 mm. internal diameter and 5 cm. length, with one end curved and attenuated into a capillary, and with a rubber sucker fixed to the other end. This pipette allowed us to take about 0·2 ml. of blood by introducing the curved capillary end behind the eye of the animal and sucking the blood. This method caused no ill effects or fatalities to the mice and many samples could be taken from the same animals on different days.

*Measurements.*—The tumours were measured by multiplying the longest diameter by the diameter at right angle to it (measured in mm.).

*Estimation of the serum glycoproteins.*—Serum glycoproteins were estimated in term of their non-glucosamine polysaccharide content. The procedure described by Shetlar, Foster and Everett (1948) was followed. The steps can be briefly summarized as follows: precipitation of the serum glycoproteins from a known dilution of serum by absolute ethanol, treatment of the precipitate with 77% sulphuric acid and addition of 1% tryptophan, boiling the mixture in a water bath for 20 minutes and then cooling. The colour developed was then read in an Evelyn photocolorimeter, using a filter of 515 m$\mu$, against standard solutions made of equal known concentrations of galactose and mannose. A blank tube contained all the chemicals, but with addition of distilled water instead of serum.

### RESULTS

### *Experiment* 1

Nineteen mice were inoculated with sarcoma 180. In order to get accurate information about the changes in the serum glycoprotein level in relation to tumour growth, we dealt with each animal separately; estimating on the same day its serum glycoprotein and measuring the size of the tumour it bore. Three of the animals were bilaterally inoculated (i.e. by two tumour implants) and 16 inoculated unilaterally.

Results are shown in Tables I, II and III.

TABLE I.—*Relation Between Serum Glycoprotein and Tumoure Size in Three of the Mice Inoculated with Sarcoma* 180, *Showing Typical Behaviour*

| | Mouse 1 | | Mouse 2 | | Mouse 3 | |
|---|---|---|---|---|---|---|
| Day | Glycoprotein (mg.%) | Tumour size (mm.$^2$) | Glycoprotein (mg.%) | Tumour size (mm.$^2$) | Glycoprotein (mg.%) | Tumour size (mm.$^2$) |
| 0 | 90 | 1 | 90 | 1 | 72 | 1 |
| 2 | — | — | 73 | 8 | — | — |
| 3 | 100 | 12 | — | — | — | — |
| 4 | — | — | — | — | 145 | 48 |
| 6 | — | 96 | — | 96 | — | — |
| 7 | 171 | 140 | 147 | 140 | 148 | 96 |
| 12 | — | — | — | — | 135 | 192 |
| 14 | 147 | 600 | 147 | 600 | 135 | 192 |
| 17 | 72 | 1200 | 75 | 1200 | Died | |
| | Died | | Died | | | |

TABLE II.—*RClation Between Serum Glycoprotein and Tumour Size in Two Mice in which Sarcoma* 180 *Spontaneously Regressed*

| | Mouse 1 | | Mouse 2 | |
|---|---|---|---|---|
| Day | Glycoprotein (mg.%) | Tumour size (mm.²) | Glycoprotein (mg.%) | Tumour size (mm.²) |
| 0 | 88 | 1 | 72 | 1 |
| 2 | 72 | 6 | — | — |
| 3 | — | — | 66 | 8 |
| 4 | — | — | 102 | 48 |
| 5 | 148 | 35 | — | — |
| 7 | — | — | 149 | 140 |
| 8 | 194 | 140 | — | — |
| 10 | — | 140 | — | 200 |
| 11 | — | 140 | — | — |
| 12 | — | — | 192 | 96 |
| 14 | 204 | 140 | 174 | 96 |
| 18 | 147 | 60 | 135 | 6 |
| 20 | 200 | — | 200 | 0 |
| 21 | 96 | 0 | — | — |

TABLE III.—*Relation Between Serum Glycoprotein and Tumour Size in Three Mice in which Sarcoma* 180 *Failed to Develop*

| | Mouse 1 | | Mouse 2 | | Mouse 3 | |
|---|---|---|---|---|---|---|
| Day | Glycoptorein (mg.%) | Tumour size (mm.²) | Glycoprotein (mg.%) | Tumour size (mm.²) | Glycoprotein (mg.%) | Tumour size (mm.²) |
| 0 | 90 | 1 | 90 | 1 | 93 | 1 |
| 1 | 72 | 1 | — | — | — | — |
| 2 | 60 | 8 | — | — | 72 | 8 |
| 4 | — | — | 102 | 1 | — | — |
| 6 | 150 | 15 | 128 | 1 | 150 | 8 |
| 8 | 300 | 8 | 300 | 0 | — | — |
| 11 | — | — | — | — | 80 | 2 |
| 14 | 205 | 2 | 192 | 0 | — | — |
| 16 | 135 | 2 | — | — | — | — |
| 20 | 100 | 0 | 95 | 0 | — | — |

In experiment 1, 14 mice developed tumours which progressively grew till they killed the animals. The serum glycoprotein in these mice underwent the following changes:

(a) A slight initial fall in most of the animals in the first two or three days,

(b) Then a gradual rise, reaching a maximum between the seventh and ninth days,

(c) Then a gradual fall which might reach normal levels in the third and fourth weeks, while the tumours were progressively growing till the death of the animals.

Table I shows the relation between serum glycoprotein level and size of tumour in three of the above group of mice.

Two mice developed tumours which grew to a good size, then the sarcoma 180 began to regress spontaneously. A higher level of serum glycoprotein was noticed in these two animals, it reached 200 mg.% and remained sustained for a time; then declined slowly until the tumours disappeared. Results are shown in Table II.

In three mice, the tumours failed to grow and in spite of this the serum glycoprotein reached very high levels—up to 300 mg.%—a level which was never reached in typically growing sarcoma 180. Results are shown in Table III.

*Experiment* 2

Twelve mice were inoculated with Ehrlich's ascites tumour; blood samples were pooled from several animals each time (about 6). Results are shown in Table IV.

TABLE IV.—*Serum Glycoprotein Levels in Mice Inoculated with Ehrlich's Ascites Tumour*

| Day | Serum glycoprotein (mg.%) |
|---|---|
| 0 . | 120 |
| 2 . | 170 |
| 6 . | 105 |
| 11 . | 115 |

Results of experiment 2 are shown in Table IV; it was noticed that there was a rise in serum glycoprotein in the early stages of growth of Ehrlich's ascites tumour, then a fall to normal levels in the late stages.

### DISCUSSION

From the above data it is evident that after inoculation of mice with sarcoma 180, the serum glycoproteins begin to rise gradually and progressively to reach a maximum between the seventh and ninth days, then decline gradually while the tumour is still increasing in size, and may fall to normal levels in the terminal stages of tumour growth. The same observations are noted during the growth of Ehrlich's ascites tumour.

Recent work has attracted much attention to the liver as the main source of glycoprotein. Greenspan (1954) demonstrated that the seromucoid level was subnormal in parenchymatous liver disease. Spiro (1958) found that the main site of synthesis of glucosamine—which is a main constituent of serum glycoproteins—was the liver and that the rate of glucosamine synthesis by the liver was much greater than by other organs such as the spleen, lungs, testicles and kidneys. Burston, Apsey and Maclagan (1965) found an increase in liver weight and perchloric acid soluble proteins in livers of rats, reaching a maximum 13 days after tumour implantation.

It might be suggested that the stimulus for rapid glycoprotein production by the liver and other organs and its elevation in the serum is the rapid proliferation of the malignant cells; perhaps through a stimulating factor released into the circulation by the growing neoplastic cells.

The role of raised serum glycoprotein level in malignancy is not clear. It might be a reaction to a non-specific stress as suggested by Boas and Peterman (1953). It might be a reaction through which the tumour tries to benefit; i.e. the tumour stimulates the production or glycoproteins in order to utilize them in its synthetic processes; this might be supported by finding higher concentrations of glycoprotein in tumours and surrounding tissues than in tissues remote from the tumours, as reported by Catchpole (1950). However the persistently high concentrations of serum glycoproteins in mice in which the tumours spontaneously regressed and in mice in which the tumours failed to grow does not seem to support this view.

Regarding the fall in serum glycoproteins which follows the initial rise; this may possibly be ascribed to failure of liver and other tissues to respond to further stimulation by the tumour. This state might represent a condition of host exhaustion which prevails in the late stages of tumour growth.

From the present work, it appears that the estimation of serum glycoproteins may have a diagnostic value in malignancy. Thus if other conditions which cause rise of serum glycoprotein level (e.g. infections) can be excluded, then the rise can be safely attributed to malignancy.

The changes in the level of serum glycoprotein observed in the present experimental work might correlate with the stage of malignant process where a rising level would be associated with an early, and a falling level with a late stage of malignancy. The application of this observation as a prognostic criterion in human malignancy does not seem readily feasible owing to the fact that human tumours generally grow at a much slower rate and attain a much smaller size in relation to body weight than animal tumours, and furthermore the patient is usually under some sort of treatment. However estimations of serum glycoproteins in patients at different stages of tumour growth and under various conditions of progression and regression seem indicated with a view to exploring its value as a prognostic criterion.

### SUMMARY

Serial estimations of serum glycoprotein levels were made in mice inoculated with sarcoma 180 and Ehrlich's ascites tumours.

The serum glycoprotein levels were found to rise gradually with the growth of the tumour to reach a peak and then to decline slowly while the tumour was still growing.

The origin as well as the cause of the initial rise and subsequent fall of serum glycoproteins in relation to tumour growth are discussed, as is the possible value of serum glycoprotein estimations in diagnosis and prognosis.

### REFERENCES

Boas, N. F. and Peterman, A. F.—(1953) *Proc. Soc. exp. Biol. Med.*, **82**, 19.
Burston, D., Apsey, M. E. and Maclagan, N. F.—(1965) *Br. J. Cancer*, **19**, 200.
Catchpole, H. R.—(1950) *Proc. Soc. exp. Biol. Med.*, **75**, 221.
Greenspan, E. M.—(1954) *Archs intern. Med.*, **93**, 863.
Harshman, S. and Bryant, G.—(1964) *Cancer Res.*, **24**, 1626.
Israel, H. L., Webster, M. B. and Maher, I. E.—(1948) *Am. J. Med.*, **6**, 754.
Macbeth, R. A. and Bekesi, J. G.—(1964) *Cancer Res.*, **24**, 2044.
Seibert, F. B., Seibert, M. V., Atno, A. J. and Campbell, H. W.—(1947) *J. clin. invest.*, **26**, 90.
Shetlar, M. R., Erwin, C. P. and Everett, M. R.—(1950) *Cancer Res.*, **10**, 445.
Shetlar, M. R., Foster, J. V. and Everett, M. R.—(1948) *Proc. Soc. exp. Biol. Med.*, **76**, 125.
Shetlar, M. R., Foster, J. V., Kelly, K. H., Shetlar, C. L. and Everett, M. R.—(1949) *Cancer Res.*, **9**, 515.
Spiro, R. C.—(1958) *J. biol. Chem.*, **234**, 742.
Weimer, H. E., Quin, F. A., Redlich-Moshin, J. and Nishihara, H.—(1957) *J. natn. Cancer Inst.*, **19**, 409.